# The Combined Use of Fractional Urate and Potassium Excretion in the Diagnosis of Diuretic-Induced Hyponatremia

**DOI:** 10.7759/cureus.15308

**Published:** 2021-05-28

**Authors:** Vincenzo Bassi, Olimpia Fattoruso

**Affiliations:** 1 Unità Operativa Complessa (UOC) di Medicina Generale e Lungodegenza, San Giovanni Bosco Hospital, Azienda Sanitaria Locale (ASL) Napoli 1 Centro, Naples, ITA; 2 Unità Operativa Complessa (UOC) di Patologia Generale, San Giovanni Bosco Hospital, Azienda Sanitaria Locale (ASL) Napoli 1 Centro, Naples, ITA

**Keywords:** siadh, siad, feua, fek, diuretics, hyponatraemia

## Abstract

Introduction

Thiazide and loop-diuretics are among the most widely used drugs in the therapy of hypertension and chronic heart failure. Furthermore, hyponatremia is the most prevalent electrolyte imbalance affecting up to 25-30% of hospitalized patients while syndrome of inappropriate antidiuresis (SIAD) is involving approximately 35% of hyponatraemic inpatients. Clinical and laboratoristic algorithms support the differential diagnosis of hypotonic hyponatremia in actual guidelines of SIAD, but a potential bias is represented by the misleading clinical assessment of the extracellular volume status in diuretic-treated patients where the necessity of withdrawal of the therapy is mandatory. We investigated the role of fractional uric acid and potassium excretion (FEUA and FEK) in the differential diagnosis of hypotonic hyponatremia in SIAD and diuretic-treated patients.

Methods

Thirty-six SIAD, 30 thiazide-induced hyponatremia (TIH), and 32 diuretic-induced hyponatremia (DIH) patients were investigated calculating FEUA and FEK values in receiver operating characteristic (ROC) curve analysis to improve the diagnostic approach of hypotonic hyponatremia.

Results

The combination of the two investigated markers showed different significative results generating patterns useful to discriminate among the three different hyponatremic groups.

Conclusion

The fractional uric acid and potassium excretion could be considered as new markers in the diagnostic approach of hyponatremic diuretic-treated patients where classical algorithms could fail.

## Introduction

Thiazide and loop-diuretics are among the most widely used drugs in the therapy of hypertension and chronic heart failure. Usually, diuretics block ion-cotransporters at different sites, inducing inhibition of tubular sodium (Na) reabsorption and an increase of urine Na and water excretion.

Hyponatremia is the most frequent electrolyte imbalance occurring in up to 25-30% of hospitalized patients and the syndrome of inappropriate antidiuresis (SIAD) is the most frequent etiology representing nearly 35% of these inpatients [1,2]. In actual guidelines of SIAD, different algorithms support the differential diagnosis of hypotonic hyponatremia [3,4], but a bias is represented by the misleading clinical assessment of the extracellular volume status in diuretic-treated patients [5,6] with the necessity of withdrawal of therapy [7]. Fenske et al. demonstrated that the fractional excretion of uric acid value (FEUA) could discriminate between SIAD and diuretic-induced hyponatremia in these confounding patients [5]. But instead, further investigations suggested that SIAD and thiazide-induced hyponatremia (TIH) showed similar FEUA values resulting in the diuretic withdrawal still mandatory to a correct diagnosis [8].

Our study aimed to investigate furtherly the role of fractional urate and potassium excretions in the differential diagnosis of SIAD and diuretic-induced hyponatremia.

## Materials and methods

Retrospectively, 143 patients, older than 18 years, presenting hyponatremia <130 mEq/L, urine osmolality >100 mEq/kg, and serum osmolality <280 mEq/kg were consecutively identified. A total of 98 patients were enrolled with the diagnosis of SIAD or diuretic-induced hyponatremia, as previously indicated [8]. Further eligibility criteria were a normal kidney, thyroid and adrenal function, sufficient dietary daily intake (at least 10 mEq/kg as solutes), no polydipsia story, and if treated, at least three months of oral diuretic therapy (hydrochlorothiazide, metolazone, indapamide, furosemide, and potassium canrenoate). The presence of liver cirrhosis and acute neurological diseases, potentially complicated by cerebral salt-wasting syndrome, was considered as a non-eligibility criterion in consideration of the interference with FEUA values [9,10]. 

Particular attention was paid to the medical and pharmacotherapy anamnesis of the enrolled patients. The effective arterial blood volume status was investigated using the parameters of change of pulse rate and blood pressure on supine and upright body position in enrolled patients [11]. Hypervolemic patients, usually affected by congestive heart failure, were identified with clinical examination of the presence of edema. The normalization of serum Na (sNa), following the withdrawal of diuretic therapy, determined the diagnosis of diuretic-induced hyponatremia. In doubtful cases, a fluid challenge test (two L isotonic saline in 24 h) was performed, and sNa increase >5 mEq/L with a ∆FENa <0.5% identified a mild hypovolemic status of a non-SIAD patient [5]. 

The laboratory parameters were tested with automated clinical analysis, using ion-selective electrodes (indirect measurement, COBAS 6000 Analyzer Series, Roche, Switzerland). A blood sample was taken in the morning, contemporary to a urine spot sample, to test Na, potassium (K), uric acid (UA), glucose, blood urea nitrogen (BUN), creatinine, and then calculate the different fractional excretions (FENa, FEUA, FEK). Laboratory tests were performed 24 h after the last oral dose of the diuretic drug.

We identified two main groups: (1) 36 patients with SIAD diagnosis, based on the classical Schwartz and Bartter criteria [12], where 12 patients (33%) were treated also with diuretics such as furosemide and/or potassium canrenoate; (2) 62 patients with hyponatremia induced by diuretics where the diagnosis was based on clinical hypovolemic or hypervolemic clinical status, normalization of hyponatremia after diuretic withdrawal, and eventual response to fluid challenge test divided into two subgroups: diuretic/no-thiazide-induced hyponatremia group (DIH, 32 patients treated with oral furosemide and/or potassium canrenoate) and thiazide-induced hyponatremia group (TIH, 30 patients treated with oral hydrochlorothiazide, metolazone, or indapamide). 

Written informed consent was obtained from all the patients before participation. The statistical analysis was performed with Prism 8.3.0 program (GraphPad Software, San Diego, USA). Means of different groups were tested with a nonparametric Kruskal Wallis test while group comparisons were performed with the Student’s test after testing for equality of variances with Levene’s test.

The accuracy of SIADH diagnosis vs. DIH and TIH groups was tested using receiver operating characteristic (ROC) analysis calculating area under the curve (AUC) by the nonparametric trapezoidal rule with 95% confidence interval (CI).

## Results

Table 1 below shows the characteristics, different etiologies, and treatments of enrolled patients.

**Table 1:** Clinical characteristics of the investigated groups. Data are mean ± standard deviation or numbers. "mdd, days" indicate the mean daily dose of diuretics and meantime of withdrawal of the diuretic therapy to obtain normonatremia in DIH and TIH groups. SIAD, syndrome of inappropriate antidiuresis; DIH, diuretic/no-thiazide-induced hyponatremia; TIH, thiazide-induced hyponatremia, M, male; F, female; AVP, arginine vasopressin analog

	SIAD group (n 36)		DIH group (n 32)	TIH group (n 30)	
Age	69.6±11.9		71.2±5.0	78.0±7.5	
Sex (M/F)	17–19		15–17	13–17	
Etiology SIAD					
Neoplasia	16		–	–	
Acute infection (pneumonia)	13		–	–	
Iatrogenic (AVP analogs)	2		–	–	
Idiopathic	5		–	–	
Extracellular volume depletion	-		16	4	
Euvolemia	42		8	21	
Extracellular volume expansion		–	8		5
No. (% of current diuretic therapy) (mdd, days)		12 (33%)	32 (100%)		30 (100%)
Furosemide (29.1 mg, 3.2 days)		7	26		–
Furosemide+canrenoate (44.2 mg + 91.6 mg, 3.0 days)		1	6		–
Hydroclorothiazide (15 mg, 4.7 days)		4	–		22
Indapamide (2.1 mg, 5.0 days)		–	–		4
Metolazone (5 mg, 3.8 days)		–	–		4

DIH and TIH patients were older compared with SIAD patients. SIAD presented different etiologies such as neoplasia (16/36 patients), iatrogenic (two of 36), acute bacterial infections, usually pneumonia (13/36), and idiopathic (five of 36). Extracellular volume depletion was a dominant finding in DIH patients vs. TIH (16/32 vs. 4/30 patients).

The fluid challenge test was performed in 31 patients resulting in 12 patients identified as SIAD, 14 patients as DIH, and five patients as TIH. BUN, sUA, and urine Na values showed significant differences between SIAD and diuretic-treated patients while FEUA was significantly different only in SIAD vs. DIH patients, showing similar FEUA values in SIAD and TIH group. But instead, FEK values were normal in SIAD while DIH and TIH patients showed increased values (Table 2).

**Table 2:** Laboratory data of the investigated groups. Data are mean (standard deviation) or numbers. *p-Value <0.05 vs. SIAD group. BUN, blood urea nitrogen; FE, fractional excretion; NS, no significative; SIAD, syndrome of inappropriate antidiuresis; DIH, diuretic/no-thiazide-induced hyponatremia; TIH, thiazide-induced hyponatremia; Na: sodium; K, potassium; UA, uric acid

	SIAD	DIH	TIH	p-Value
Serum				
Na (135-145 mEq/L)	126.1 (7.1)	128.7 (4.2)	126.4 (6.0)	NS
K (3.5-4.5 mEq/L)	4.2 (0.5)	4.6 (1.0)	4.0 (1.2)	NS
Creatinine (0.5-1.2 mg/dL)	0.5 (0.2)	1.1 (0.4)	0.9 (0.4)	NS
BUN (20-50 mg/dL)	23.7 (10.4)	64.0 (22.8)*	50.1 (31.6)*	0.01
UA (3.4-7.0 mg/dL)	2.5 (1.1)	6.8 (1.9)*	4.3 (2.2)*	0.001
Osmolality (275-285 mEq/kg)	254.4 (13)	266.9 (11)	260.0 (14)	NS
Urine				
Na (50-200 mEq/L)	99.5 (68.6)	31.0 (17.1)*	52.2 (43.4)*	0.001
K (25-100 mEq/L)	34.6 (7.7)	67.0 (26.0)	42.4 (15.1)	NS
Osmolality (50-1200 mEq/kg)	494.3 (274)	537.6 (170)	470.0 (189)	NS
FE				
Na (<1%)	1.0 (0.9)	0.4 (0.3)	1.2 (0.6)	NS
UA (5-9%)	16.2 (5.0)	5.7 (2.1)*	17.5 (8.2)	0.001
K (8±2%)	6.1 (2.7)	11.2 (6.7)*	14.2 (7.1)*	0.01

**Figure 1:**
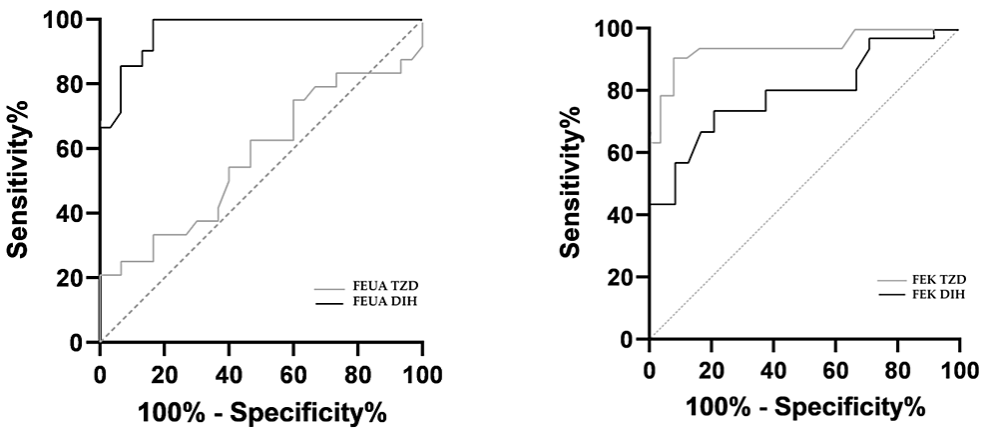
ROC curves analysis of FEUA (left) and FEK (right) values of SIAD vs. DIH and TZD groups. AUC: 95% confidence interval; FEUA SIAD/DIH: AUC 0.96 (0.92-1.0); SIAD/TZD: AUC 0.58 (0.41-0.73); FEK SIAD/DIH: AUC 0.79 (0.67-0.91); SIAD/TZD: AUC 0.94 (0.87-1.0) SIAD, syndrome of inappropriate antidiuresis; DIH, diuretic/no-thiazide-induced hyponatremia; TIH, thiazide-induced hyponatremia; AUC, area under the curve, FEUA, fractional excretion of uric acid; FEK, fractional potassium excretion; ROC, receiver operating characteristic; TZD, thiazide

ROC analysis confirmed that FEUA discriminated significantly between SIAD and DIH patients (AUC 0.96, <0.001) while was a poor marker in SIAD vs. TIH patients (0.58, no significative {NS}). Adversely, FEK discriminated significantly among SIAD vs. both DIH (AUC 0.80 <0.001) and TIH (0.94, <0.0001) groups (Figure 1).

**Figure 2:**
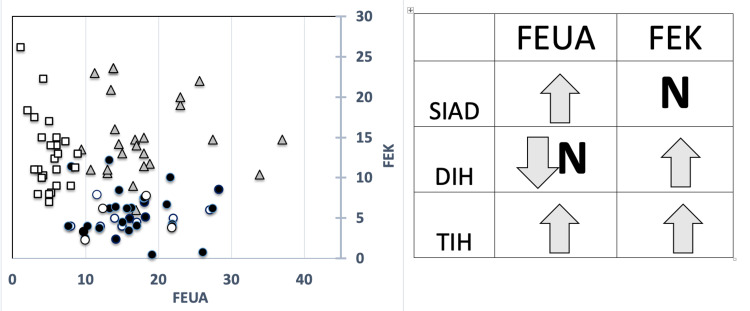
distribution and different patterns of FEUA and FEK values On the left, distribution of FEUA and FEK values in the three different investigated groups, TIH (gray triangles); DIH (white squares), and SIAD patients with (white dots) and without (black dots) diuretic treatment. On the right, the different patterns of FEUA and FEK values in the three different investigated groups. N, normal range; SIAD, syndrome of inappropriate antidiuresis; DIH, diuretic/no-thiazide-induced hyponatremia; TIH, thiazide-induced hyponatremia; FEUA, fractional excretion of uric acid; FEK, fractional excretion of potassium

Thus, the different patterns of FEK/FEUA combination (SIAD: high FEUA/normal FEK, DIH: normal-reduced FEUA/high FEK, TIH: high FEUA/high FEK value) were able to discriminate among the three investigated groups (Figure 2). The coexistence of diuretic therapy in SIAD patients did not affect the diagnostic accuracy of the different patterns of fractional excretion (FEUA: 15.1%±7.3, FEK: 5.7%±3.7) (Figure 2).

## Discussion

Hyponatremia is by far the most frequent electrolyte imbalance found in the hospital population with SIAD representing nearly 35% among the different etiologies [2]. Usually, the diagnosis of hypotonic hyponatremia is based on clinical and laboratoristic algorithms where the natriuretic effect, induced by diuretic therapy, could represent a confounding role. Usually, thiazides, in the diuretic group, are at high risk to reduce serum sodium levels increasing six-fold the risk vs. no exposed patients with an estimated incidence of 11% of hyponatremia in the geriatric population [13].

TIH could be dependent, beyond on natriuresis [14], on water retention induced by an increase of arginine vasopressin activity and/or upregulation of aquaporin-2 expression, resembling a laboratory pattern of SIAD [15]. Another possible key to explain TIH onset could be identified in the different gene expressions of specific prostaglandin transporters in patients prone to develop hyponatremia [16,17]. Furthermore, also hypokalaemia, a frequent hallmark in thiazide-treated patients, increases the volume receptor-dependent release of vasopressin [18]. Anyway, our patients presented a normokalemia, probably in consideration of the low daily dose of diuretic therapy.

Commonly, TIH patients are clinically euvolemic with an extracellular volume expansion similar to SIAD [19]. Then, in order to obtain a correct diagnosis, the withdrawal of diuretics is considered mandatory in the real world resulting in a longer hospitalization period and increased hospital admission costs.

Serum uric acid (sUA) and its fraction excretion are valuable markers to identify the volemic status in acute patients. The renal handling of uric acid is exclusively in the proximal tubule, preserved by interferences induced by the most used diuretics [20]. Moreover, fractional excretions on a urine spot sample represent an accurate parameter avoiding 24 h urine collection [21]. SIAD is generally associated with sUA level <4 mg/dL and the relative increase in FEUA value >12% [5] dependent on a decrease in urate tubular reabsorption [22] whereas secretion seems to be appropriate for the level of uricemia [23]. Fenske et al. showed that FEUA was a reliable marker to discriminate SIAD vs. diuretic-induced hyponatremia patients at admission, but a bias of this study was that only 13% of enrolled patients were treated with thiazide therapy [5] and a recent study has suggested that FEUA value does not identify correctly SIAD vs. TIH patients [8]. Our data confirmed that FEUA is a poor marker in the differential diagnosis of SIAD vs. TIH patients, probably in consideration that the bulk of TIH patients shows a phenotype of euvolemia resembling SIAD pattern (in our study 22/30 patients, 73%).

Usually, an increased FEK value in hypotonic hyponatremia is a typical marker of diuretic therapy or renal salt-wasting (RSW) syndrome. In our investigated patients, FEK was useful to discriminate among SIAD (normal FEK values) vs. DIH and TIH patients (increased FEK values). Unexpectedly, normal FEK values were observed also in SIAD patients on diuretic therapy. We suggest that the increased glomerular filtration rate as a result of extracellular fluid volume expansion in SIAD induces an increased filtered K in the glomerulus with a normal FEK value. Moreover, the described euvolemic/mild hypervolemic status in SIAD decreases aldosterone levels reducing the quantity of K lost at the renal tubules.

Furthermore, normal FEK values found in our hypotonic hyponatremia patients confirm that RSW compared to SIAD is a rare cause of hyponatremia.

## Conclusions

In conclusion, the pattern of association of FEK/FEUA values in hypotonic hyponatremia could represent a further improvement in the differential diagnosis of SIAD vs. hyponatremia induced by diuretics. Anyway, further studies with larger cohorts of patients are necessary to confirm the effective diagnostic role of these parameters in hypotonic hyponatremia.
